# Multi-Lens Arrays (MLA)-Assisted Photothermal Effects for Enhanced Fractional Cancer Treatment: Computational and Experimental Validations

**DOI:** 10.3390/cancers13051146

**Published:** 2021-03-08

**Authors:** Hyejin Kim, Hanjae Pyo, Hyeonsoo Kim, Hyun Wook Kang

**Affiliations:** 1 Industry 4.0 Convergence Bionics Engineering, Pukyong National University, Busan 48513, Korea; hyejink@pukyong.ac.kr (H.K.); hanjaepyo@pukyong.ac.kr (H.P.); hyeonsookim@pukyong.ac.kr (H.K.); 2 Department of Biomedical Engineering, Pukyong National University, Busan 48513, Korea

**Keywords:** fractional laser therapy, photothermal therapy, micro-lens array, colon cancer, cancer treatment

## Abstract

**Simple Summary:**

Colorectal cancer is one of the most common cancers and the third leading cause of cancer-related deaths in the United States. As a non- or minimally invasive cancer treatment, photothermal therapy (PTT) has been widely used to generate irreversible thermal injuries in tumors. However, conventional PTT employs an end-firing flat fiber to deliver laser energy, leading to the incomplete removal of tumor tissues due to an uneven beam distribution over the tumor surface. Multi-lens arrays (MLA) generate multiple micro-beams to uniformly distribute laser energy on the tissue surface. Therefore, the application of MLA for PTT in cancer affords a spatially enhanced distribution of micro-beams and laser-induced temperature in the tumor. The purpose of the current study is to computationally and experimentally demonstrate the therapeutic benefits of MLA-assisted fractional PTT on colorectal cancer, in comparison to flat fiber-based PTT.

**Abstract:**

Conventional photothermal therapy (PTT) for cancer typically employs an end-firing flat fiber (Flat) to deliver laser energy, leading to the incomplete treatment of target cells due to a Gaussian-shaped non-uniform beam profile. The purpose of the current study is to evaluate the feasibility of multi-lens arrays (MLA) for enhanced PTT by delivering laser light in a fractional micro-beam pattern. Computational and experimental evaluations compare the photothermal responses of gelatin phantoms and aqueous dye solutions to irradiations with Flat and MLA. In vivo colon cancer models have been developed to validate the therapeutic capacity of MLA-assisted irradiation. MLA yields 1.6-fold wider and 1.9-fold deeper temperature development in the gelatin phantom than Flat, and temperature monitoring identified the optimal treatment condition at an irradiance of 2 W/cm^2^ for 180 s. In vivo tests showed that the MLA group was accompanied by complete tumor eradication, whereas the Flat group yielded incomplete removal and significant tumor regrowth 14 days after PTT. The proposed MLA-assisted PTT spatially augments photothermal effects with the fractional micro-beams on the tumor and helps achieve complete tumor removal without recurrence. Further investigations are expected to optimize treatment conditions with various wavelengths and photosensitizers to warrant treatment efficacy and safety for clinical translation.

## 1. Introduction

Colorectal cancer (CRC) is the fourth most common cancer in women and the fifth most common cancer in men, and is attributed to poor diet, smoking, excessive drinking, and obesity [1]. It is also the third leading cause of cancer-related deaths in the United States. Most CRCs develop from polyps in the colon, which are easily removable, but if left untreated, can change into malignant cancer with time [2]. Despite conventional treatments, such as radio- and chemotherapy, CRC remains associated with high mortality and recurrence as a result of incomplete treatment of the tumor region [3,4]. Therefore, the development of a novel therapeutic modality for CRC is pivotal in advancing clinical outcomes and improving treatment safety.

Photothermal therapy (PTT) has been widely studied as a non- or minimally invasive and effective cancer treatment. The primary purpose of applying PTT is to locally deposit volumetric heat in tumor tissue upon light absorption, leading to irreversible thermal injury, cell apoptosis, and necrosis [5]. Photothermal agents are often used to selectively absorb incident light and confine the induced thermal effects specifically to the targeted tissue. A number of research studies have reported various nanomaterials to enhance photothermal effects during tumor treatment. For instance, the application of gold nanoparticles can induce a temperature increase in the tissue in terms of exclusive surface modifications and high light absorption at a wavelength of 532 nm [6,7,8]. Functionalized carbon nanomaterials have been widely investigated as drug delivery vehicles, biomedicine, and photothermal agents [9,10,11]. A bare-cut flat fiber (Flat) is typically employed to deliver laser light to tumor tissue for PTT. However, owing to a Gaussian beam profile, tumors with a three-dimensional irregular shape often experience non-uniform distributions of light and laser-induced temperature during Flat-based irradiation [12]. Consequently, the inhomogeneous temperature in the tumor weakens therapeutic effects and eventually leads to the incomplete treatment of target cancer cells. Hence, the remaining cancer cells can regrow, thereby causing the tumor to recur, which decreases the survival rate.

Laser treatments in dermatology often employ multi-lens arrays (MLA) for the delivery of fractional laser light to uniformly distribute micro-beams on the skin tissue. Fractional laser treatment with MLA is a minimally invasive treatment method that entails microscopic thermal lesions on the tumor with minimal thermal injury to surrounding tissue [13]. MLA delivers multiple micro-beams at high fluences, whereas Flat transmits a single macro-beam at low fluences [14]. Thus, unlike Flat-based irradiation, MLA-assisted irradiation can accompany wide and uniform distributions of the temperature in the tissue upon light absorption. In fact, fractional skin treatment with micro-beams can accelerate skin rejuvenation and the recovery of the treated skin by preserving healthy surrounding tissue, compared to the flat-beam skin treatment [15].

The aim of the present study was to demonstrate the therapeutic capacity of MLA-assisted fractional laser treatment in tumors, and compare it to conventional Flat-based laser treatment. We hypothesized that the MLA-assisted irradiation provides spatially wide and uniform temperature profiles in the tumor to effectively eradicate cancer cells by generating microscopic thermal lesions in the tissue. Continuous laser irradiation in conjunction with a photothermal agent was employed to augment the coupling efficiency and conversion of optical into thermal energy during PTT [16]. The proposed treatment method was validated theoretically and experimentally to confirm laser-induced thermal responses of in vivo tumor models to MLA-assisted irradiation. Histological analysis was performed to assess the degree of thermal damage to warrant treatment efficacy and safety of the proposed MLA-assisted PTT.

## 2. Materials and Methods

### 2.1. Light Source

**Figure 1:**
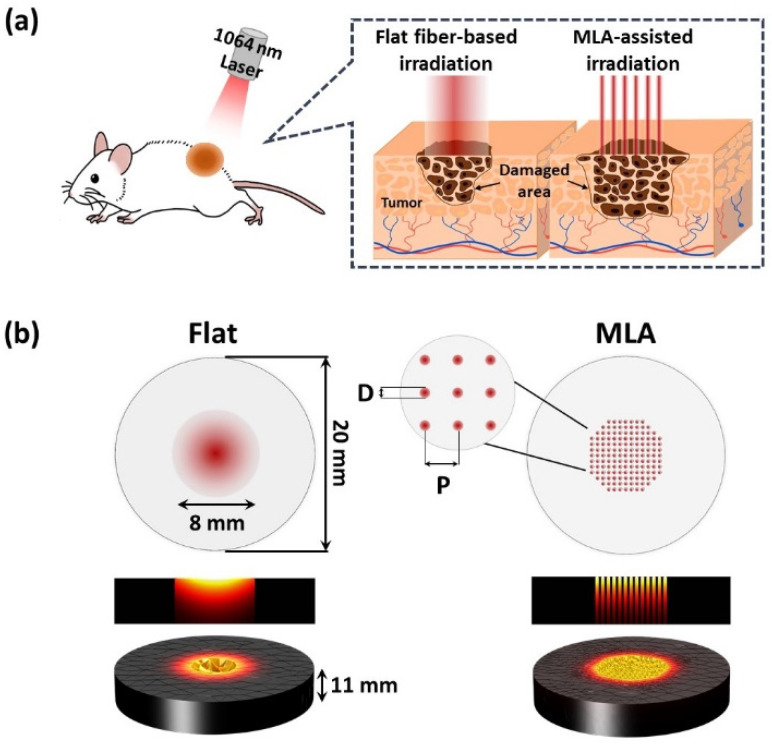
Schematic representations of (**a**) laser irradiation with flat fiber (Flat) and micro-lens arrays (MLA) on in vivo tumor model and (**b**) geometry of gelatin phantom (20 mm in diameter and 11 mm in thickness) for numerical simulation of laser irradiation with Flat and MLA (D = micro-beam diameter of 350 μm; P = distance between two consecutive micro-beams of 500 μm).

This study employed a 1064 nm laser system (FC-W-1064B-30W, CNI, Changchun, China) to induce photothermal effects. A 600-µm multimode end-firing flat fiber (Flat; multimode optical fibers; 600 µm core diameter, Thorlabs Inc., Newton, NJ, USA) and multi-lens arrays (MLA; fused silica; focal length = 40 mm; 145 micro-beams; micro-beam diameter = 350 μm; macro-beam diameter = 8 mm; Bluecore company, Busan, Republic of Korea) were used to deliver laser light (Figure 1a). For direct comparison, an identical beam diameter of 8 mm was applied for both Flat-based and MLA-assisted irradiation. The applied power ranged from 0.5–1.5 W (irradiance = 1–3 W/cm^2^), and the irradiation time was 180 s. A power detector (PD-300-3W, Ophir, Jerusalem, Israel) in conjunction with a power meter (Nova II, Ophir, Jerusalem, Israel) measured the laser power from Flat and MLA before and after each test to replicate identical experimental conditions.

### 2.2. Temperature Assessments

We initially performed numerical simulations on the photothermal responses of gelatin phantoms to compare the effects of Flat and MLA on spatial distributions of temperature and experimentally validated the simulation results. IR 1061 dye was used as a chromophore to absorb 1064 nm laser light for thermal assessment.

#### 2.2.1. Numerical Simulation

Numerical simulations were conducted to predict instant thermal responses of gelatin phantoms mixed with IR 1061 dye to laser irradiations with Flat and MLA using COMSOL software (5.3, COMSOL Multiphysics, COMSOL Inc., Burlington, MA, USA). Figure 1b displays the geometry of the phantom model (20 mm in diameter and 11 mm in thickness). A 1-W 1064 nm laser light was perpendicularly irradiated on the top surface of the phantom for 15 s. Flat delivered a Gaussian beam distribution with a diameter of 8 mm in a single beam spot, whereas MLA had 145 uniformly distributed micro-beams in an 8 mm macro-beam spot (micro-beam diameter = 350 μm and distance between two consecutive micro-beams = 500 μm). The corresponding irradiance was 1 W/cm^2^. The volumetric heat generation (Q, W/cm^3^) induced by light absorption is described as follows [17]:

Q = µ_a_·I(r,z), (1)

where µ_a_, I, r, and z are the absorption coefficient (mm^-1^), fluence rate (W/cm^2^), radial distance (mm), and depth (mm), respectively. For the Flat irradiation, I(r,z) is described as follows:

I(r,z) = P/(π·r_0_^2^)·exp(−µ_a_·z) (2)

where P and r_0_ are the laser power (W) and irradiated beam radius (mm), respectively. For the MLA irradiation, I(r,z) reflected uniform distributions of multiple micro-beams on the phantom surface (rθ-plane; Figure 1b) as follows:

I(r,z) = P/(π·r_0_^2^)·exp(−r^2^/r_0_^2^)·exp(−µ_a_·z) (3)

The Pennes’ bio-heat transfer equation was then used to describe temperature development in the phantom model [17]:

(4) $$\rho C_{P}\;\cdot \;\partial T/\partial t\;+\;\rho C_{P}\;\cdot \;\vec{u}\;\cdot \nabla T\;=\;\nabla \cdot \left(k\nabla T\right)\;+\;Q$$

where ρ, C_p_, T, t, $\;\vec{u}\;$, and k, are the density of the phantom (kg/m^3^), specific heat for the phantom (J/kg·K), temperature (K), time (s), normal vector for heat transfer, and thermal conductivity of the phantom (W/m·K). The initial temperature of the gelatin phantom was set to 5 °C. Convective heat transfer was applied on the top surface of the phantom using the following equation [17]:

(5) $$-\vec{n}\cdot \left(-k\nabla T\right)\;=\;h\left(T\;-\;T_{\mathrm{air}}\right)$$

where **n**, h, and T_air_ are normal vectors of heat flux, convective coefficient (W/m ·K), and ambient air temperature (20 °C). The rest of the phantom surfaces were assumed to be insulated (**n**·k·∇T = 0 according to Neumann boundary condition). Table 1 summarizes all the physical properties used in the numerical simulations.

#### 2.2.2. Phantom Evaluations

Gelatin-based phantoms were fabricated and tested to validate numerical simulations of the spatial distributions of temperature after Flat-based and MLA-assisted irradiation. Gelatin powder (10% [*w*/*v*], Sigma Aldrich, St. Louis, MO, USA) was mixed with distilled water at 70 °C until the powder completely melted. Then, 0.03% (*w*/*v*) IR 1061 dye (Sigma Aldrich, St. Louis, MO, USA) was added to the prepared mixture as a chromophore to absorb the incident 1064 nm laser light. The final mixture was poured into a six-well culture plate, and the plate was stored at 5 °C overnight to achieve sufficient solidification. The 1064 nm laser light was irradiated at 1 W/cm^2^ for 15 s with Flat and MLA on the prepared phantoms (20 mm in diameter and 11 mm in thickness). Each test was repeated four times (*n* = 4). Both the top surface and cross-section of each treated phantom were photographed to assess the extent of gelatin removal as a result of Flat-based and MLA-assisted photothermal interactions. Image J (National Institute of Health, Bethesda, MD, USA) was used to measure the physical dimensions of all ablated craters and to estimate the corresponding ablation volume for quantitative comparison.

### 2.3. Aqueous Solution Experiments

To identify the appropriate treatment conditions for in vivo testing, we tested four different concentrations of IR 1061 dye in PBS (0, 100, 200, and 300 µg/mL in PBS) in 24-well cell culture plates with three different irradiances (1, 2, and 3 W/cm^2^ and irradiation time = 180 s). The solutions were poured into 24-well cell culture plates (volume in each well: 500 µL). A 1064 nm laser light was irradiated perpendicularly onto the solution surface by means of Flat and MLA. Each test was repeated four times (*n* = 4). A thermal imaging infrared (IR) camera (A325, FLIR, Wilsonville, OR, USA) was used to monitor the spatiotemporal development of the temperature on the solution surface during the laser irradiation. After 180-s of irradiation, IR images were captured from the two conditions. The temperatures at the central (T_C_) and peripheral (T_P_) regions were measured from IR images for direct comparison. T_C_ represents the maximum temperature after laser irradiation, and T_P_ indicates the temperature at the outmost boundary of the beam spot (r = 4 mm). Based on the dosimetry findings, in vivo experiments selected the following conditions: dye concentration = 300 µg/mL in PBS: laser irradiance = 2 W/cm^2^ and irradiation time = 180 s).

### 2.4. In Vivo Validations

CT26 murine colon cancer cells were used to fabricate in vivo tumor models for the comparison of both irradiation modes. CT26 cells were obtained from the Korean Cell Line Bank and cultured in Dulbecco’s modified Eagle’s medium (DMEM, Corning, NY, USA) with 10 % fetal bovine serum (FBS, Corning, NY, USA) and 1 % antibiotic-antimycotic (Gibco, Grand Island, NY, USA). The prepared cancer cells were kept in a humidified incubator at 37 °C in a 5 % CO_2_ atmosphere.

In total, 12 BALB/c female mice (age: 5 weeks; weight: 20–25 g) were procured from Hana Biotech (Suwon, Korea) to create tumor models. All animals were individually housed in a pathogen-free cage at the Animal Research Centre facility under standard conditions. The mice were acclimated for a week, and subsequently, the prepared CT26 cancer cells (3 × 10^5^ cells) were injected into the back of each mouse and incubated for one week to grow the tumors. Each mouse was randomly sorted into three groups for comparison (*n* = 4 per group): control, Flat, and MLA. Before conducting the experiments, the mice were anesthetized in a chamber using a respiratory anesthesia system (Classic T3, SurgiVet, Minneapolis, MN, USA) with 3 % isoflurane (Terrell^TM^ isoflurane, Piramal Critical Care, Bethlehem, PA, USA) in oxygen (0.6 L/min). A week after injection of the cancer cells, diluted IR 1061 dye (concentration = 200 µg/mL in PBS) was administered into each animal intra-tumorally to selectively absorb the incident 1064 nm laser light for PTT. Then, two hours after the dye injection, the tumor-bearing models were irradiated with 1064-nm laser light at 2 W/cm^2^ for 180 s (selected from solution experiments) using Flat and MLA. The current study applied a single treatment to all animals. A thermal imaging IR camera was used to monitor temperature elevations in the tumor during laser irradiation. Thereupon, all the treated tumors were monitored and photographed using a digital camera (D5100, Nikon, Tokyo, Japan) at four different time points: 0 (D 0), 3 (D 3), 7 (D 7), and 14 (D 14) days after laser treatment. Image J was used to estimate tumor size by measuring its length (*l* in mm) and width (*w* in mm). The tumor volume (*V* in mm^3^) was calculated using the formula *V = (l × w^2^)/2* [21]. All animal experimental procedures were implemented in accordance with a standard experimental setup following the Korean National Institutes of Health (KNIH) guidelines. The protocol was approved by the Institutional Animal Care and Use Committee at Pukyong National University (Permit Number: PKNUIACUC2019-30).

### 2.5. Histological Analysis

To evaluate the in vivo photothermal treatment efficacy, all the treated tumor-bearing mice were euthanized in a chamber with an overdose of CO_2_ gas for 14 days (D 14) after laser irradiation with Flat and MLA. The tumor tissue samples were harvested aseptically and fixed in 10% neutral formalin solution (Sigma Aldrich, St. Louis, MO, USA) for three days. After fixation, each sample was sectioned at 4–5 µm for slide preparation. The prepared sections were stained with standard hematoxylin and eosin (HE) and TUNEL staining assay (TumorTACS In situ Apoptosis Detection Kit, R&D Systems Inc., Minneapolis, MN, USA) to confirm the extent of irreversible thermal coagulation in the treated tumor tissue. All histological slides were photographed using optical microscopy (20X for HE and 100X for TUNEL; Motic easyscan, Motic, Kowloon, Hong Kong, China). Subsequently, a pathologist conducted gross examinations on the histology slides of three groups (control, Flat, and MLA; *n* = 4 per group) and scored the histological responses of the random spots in the slides semi-quantitatively [22,23] in a score range between 1 and 3, where 1 (absent or minimal) represents no or minimal thermal damage to tumor tissue and negative response of TUNEL staining (blue-green color); 2 (moderate) represents mild cellular shrinkage resulting from laser-induced injury and positive reaction of TUNEL staining (brown color); 3 (severe) represents severe thermal injury induced by the laser irradiation and positive reaction of TUNEL staining (brown color) in the same area.

### 2.6. Statistical Analysis

All data are expressed as mean ± standard deviation for four independent experiments. Statistical analysis was performed using SPSS software 22 (SPSS Inc., Chicago, IL, USA). For nonparametric statistical analysis, the Kruskal–Wallis (KW) and Mann–Whitney U tests with Bonferroni correction (MU) were performed to compare multiple and two groups, respectively, and statistical significance was considered at *p* < 0.05.

## 3. Results

### 3.1. Temperature Assessments

**Figure 2:**
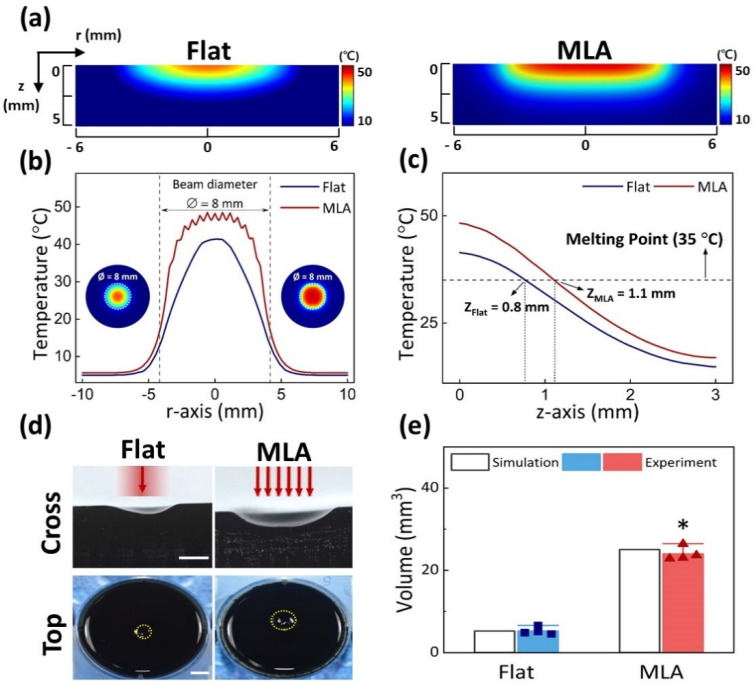
Comparative thermal evaluations on gelatin phantom models between flat fiber (Flat) and MLA: (**a**) cross-sectional (rz-plane) temperature distributions from numerical simulations, (**b**) temperature profiles at surface along r-axis (acquired from (**a**)), (**c**) axial temperature distributions along z-axis from center of irradiated area in (**a**), (**d**) experimental validations in gelatin phantom models after irradiation with 1064 nm laser light using Flat and MLA (1 W/cm^2^ for 15 s; 15 J/cm^2^; yellow dotted lines = ablated area; scale bar = 5 mm in cross-sectional images and 2 mm in top-view images), and (**e**) quantitative comparison of ablated volume between Flat and MLA irradiations from simulations and experiments (*n* = 4; * MU *p* < 0.005 vs. Flat). Note that dashed lines in (**c**) represent the melting point (35 °C) of the gelatin phantom model.

Numerical simulations and experimental validations were performed to estimate the temperature distributions after 180 s laser irradiation with Flat and MLA (Figure 2). Figure 2a presents cross-sectional temperature distributions in gelatin phantom models after 180 s irradiation with Flat (left) and MLA (right) from the simulations. Under the same irradiation conditions, the Flat-based irradiation showed a narrower and shallower profile of the laser-induced temperature. In contrast, the MLA-assisted laser irradiation entailed a deeper and wider distribution of the laser-induced temperature in the simulation model. Radial distributions of the surface temperature (obtained from Figure 2a) demonstrate that MLA induced a 7.1 °C higher peak temperature in a flatter top profile, compared to Flat (Figure 2b). Axial temperature distributions (obtained from Figure 2a) confirm that MLA induced a higher temperature distribution along the z-axis with a higher surface temperature than Flat (Figure 2c). The extent of thermal deformation in the gelatin phantom was estimated by considering the melting point of the phantom at 35 °C (dashed line). According to Figure 2c, MLA produced a 1.4-fold deeper deformation compared to Flat (i.e., z_Flat_ = 0.8 mm vs. z_MLA_ = 1.1 mm, corresponding to 35 °C). Figure 2d displays cross-sectional and top-view images of the gelatin phantoms irradiated with Flat and MLA. Compared to Flat, MLA created a deeper and wider ablation profile. The ablated phantom volumes were quantitatively compared between Flat and MLA (Figure 2e). MLA ablated a five-fold larger ablation volume than Flat (4.9 ± 1.1 mm^3^ for Flat vs. 24.9 ± 2.5 mm^3^ for MLA; *p* < 0.05). Both numerical simulations and experimental validations are in good agreement in the estimated volumes.

### 3.2. Dosimetry Tests

**Figure 3:**
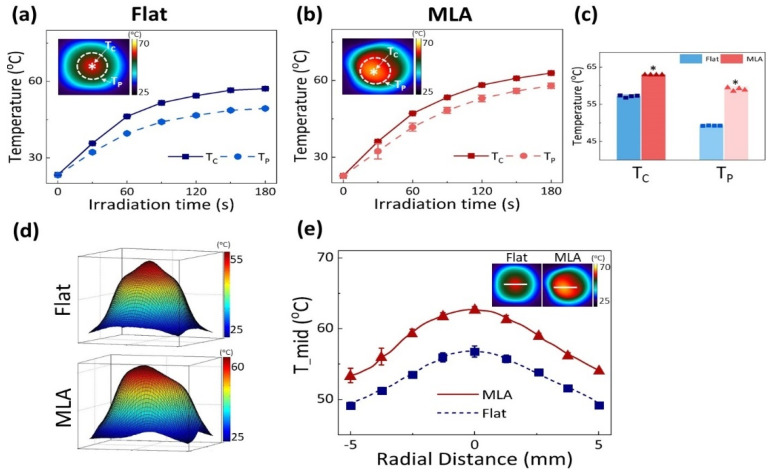
Comparison of photothermal effects between Flat-based and MLA-assisted irradiations at 2 W/cm^2^ for 180 s on IR 1601 aqueous solution (300 μg/mL in PBS): temporal developments of surface temperature at central (T_C_; measured at r = 0 mm; white asterisk) and peripheral (T_P_; measured at r = 4 mm; white dashed line) regions obtained from (**a**) Flat and (**b**) MLA, (**c**) comparison of elevated temperatures at T_C_ and T_P_, (**d**) 3D images of temperature fields after irradiation (360 J/cm^2^), and (**e**) comparison of radial temperature distributions along middle line (T__mid_) of IR images. Note that the inlets in (**a**), (**b**), and (**e**) represent the captured IR images after 180 s irradiation (*n* = 4; * MU *p* < 0.005 vs. Flat).

To identify the appropriate conditions for in vivo laser treatment, 1064 nm laser light with Flat and MLA was tested on an IR 1061 aqueous solution at various laser irradiances and concentrations (Figure S1). The maximum temperature increases up to 38.5 °C for Flat and 44 °C for MLA at 3 W/cm^2^ irradiance and dye concentration of 300 µg/mL. As the tissue temperature reaches 60 °C, collagen and protein denaturation commence, leading to irreversible thermal coagulation. Thus, considering the initial temperature of in vivo tissue (~37 °C), we selected an irradiance of 2 W/cm^2^ and a dye concentration of 300 µg/mL to warrant photothermal effects in in vivo experiments. The temperatures at the central (T_C_) and peripheral (T_P_) regions on the solution surface were measured and compared between Flat and MLA (Figure 3a,b). Regardless of the irradiation method, the temperatures (T_C_ and T_P_) gradually increased with irradiation time, and T_C_ was higher than T_P_ because of spatial distributions of the incident laser beam. Notably, the difference between T_C_ and T_P_ was evidently smaller in MLA (~8%) than in Flat (~16%) because of the uniform distributions of the micro-beams. At 180 s irradiation time, MLA yielded a higher T_C_ (56.9 °C for Flat vs. 62.9 °C for MLA) and T_P_ (49.4 °C for Flat vs. 57.9 °C for MLA, *p* < 0.005; Figure 3c). IR imaging confirmed rapid and widespread developments of the surface temperatures during the MLA-assisted irradiation, compared to the Flat-based irradiation (Figure S2). Figure 3d displays the 3D temperature profiles acquired from the IR images after 180 s irradiation. MLA exhibited a relatively flat top temperature distribution than Flat that showed a Gaussian distribution. Similar to Figure 1b, the radial distributions of the solution temperature confirmed that MLA was accompanied by an approximately 5.2 °C higher temperature increase along with a 29 % wider distribution, compared to Flat (Figure 3e). It should be noted that the peak temperature from MLA was slightly off-center because of oblique IR imaging to have a full view of the irradiated area and to avoid any thermal damage during the irradiation.

### 3.3. In vivo Treatment

**Figure 4:**
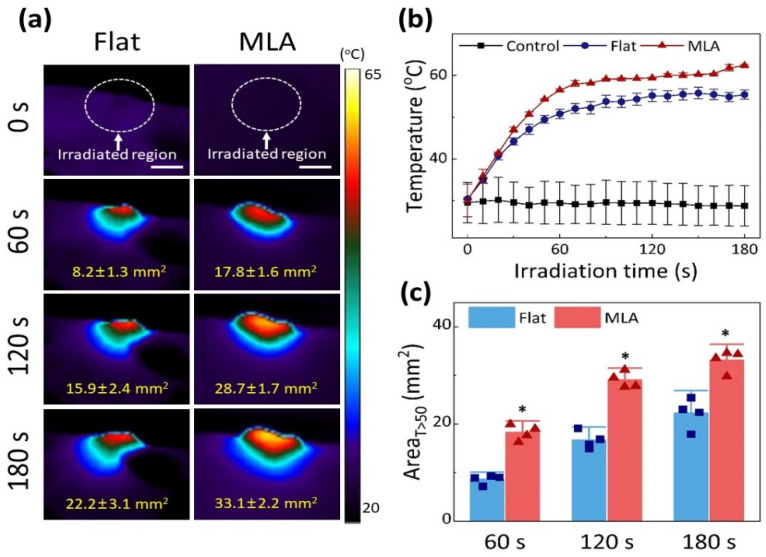
In vivo thermographic measurements with Flat-based and MLA-assisted irradiations at 2 W/cm^2^ irradiance for 180 s (dye concentration = 300 μg/mL in PBS): (**a**) IR images of CT26 tumor-bearing mouse model captured at various irradiation times, (**b**) temporal developments of maximum temperature measured from irradiated area in tumor (white dashed lines in (**a**)), and (**c**) comparison of treatment areas with temperature of 50 °C or higher (T > 50) at three irradiation times (*n* = 4 per group; scale bar = 5 mm; * MU *p* < 0.005 vs. Flat).

Figure 4a presents IR images of tumor regions treated at various times from the Flat (left) and MLA (right) groups (2 W/cm^2^ for 180 s; 360 J/cm^2^). All groups showed a temperature increase in the tumor during laser irradiation. At 180 s, the MLA irradiation exhibited a higher maximum temperature in a wider thermal region than the Flat irradiation. Figure 4b compares the temporal elevations of the peak temperature at the tumor surface. Both Flat and MLA irradiations demonstrated that the tumor temperature initially increased with the irradiation time but became saturated around 60 s after irradiation. At the end of the irradiation, MLA reached a higher maximum temperature than Flat (55.2 °C for Flat vs. 62.4 °C for MLA; *p* < 0.005). The treated regions with a temperature of 50 °C or higher were also compared between the two groups at various times (Figure 4c). Regardless of irradiation time, MLA created around 1.5-fold larger regions than Flat, implicating a wider temperature distribution attributed to the uniform delivery of micro-beams (i.e., 22.2 ± 3.1 mm^2^ for Flat vs. 33.1 ± 2.2 mm^2^ for MLA at 180 s; *p* < 0.005).

**Figure 5:**
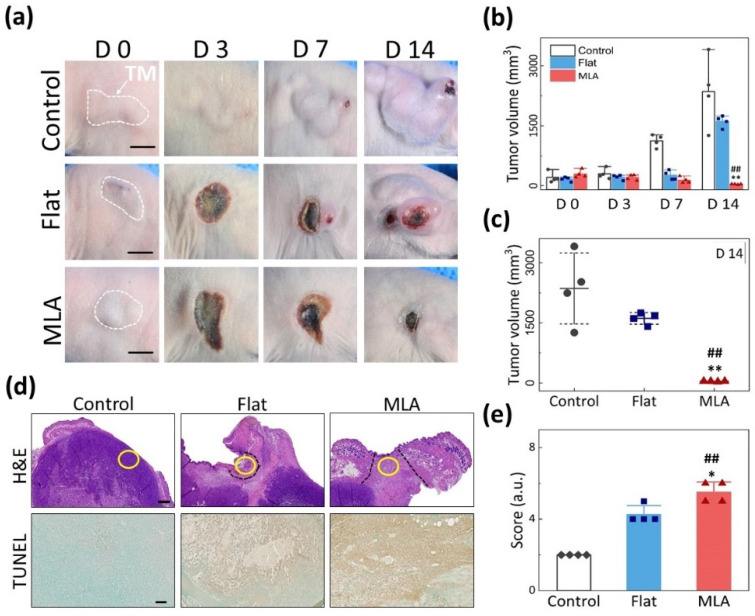
In vivo photothermal treatment of CT26 tumor-bearing mouse models with Flat-based and MLA-assisted irradiations at 2 W/cm^2^ for 180 s (dye concentration = 300 μg/mL in PBS): (**a**) compilation of images of tumor model at various time points after laser irradiation (TM = tumor region; scale bar = 5 mm), (**b**) comparison of tumor growth after laser treatment, (**c**) statistical comparison of tumor volumes at D 14, (**d**) HE-stained (top row; 20× scale bar = 600 µm) and TUNEL-stained (bottom row; 100× scale bar = 50 µm) images of treated tumor cross-sections at D 14, and (**e**) semi-quantitative evaluations of histopathological responses from treated tumor tissue. Note that black dashed and yellow solid lines in (d) represent the laser-treated area and the observed area for TUNEL analysis, respectively (*n* = 4 per group; KW *p* < 0.01; ^##^ MU *p* < 0.005 vs. control; ** MU *p* < 0.005 and * MU *p* < 0.05 vs. Flat).

All treated tumors were monitored for 14 days to characterize the treatment efficacy of Flat and MLA irradiations (Figure 5). According to Figure 5a, the tumor size from the control (no treatment) slightly increased for three days after the treatment but showed a significant increase afterwards. The Flat group maintained the tumor size for seven days, but the tumor rapidly increased with a scab on the treated surface. On the contrary, the MLA group had a slightly larger treated lesion (D 0), and the tumor size noticeably decreased over time. Figure 5b compares the quantified tumor volumes at various times among the three groups. Evidently, the MLA group exhibited a continuous reduction in the tumor size with healing time, unlike the control and Flat groups. At D 14, the tumor from the MLA group was almost eradicated (*p* < 0.005 vs. control and Flat; Figure 5c). Figure 5d presents histological images of the treated tumor tissue at D 14 to validate the antitumor effects of Flat and MLA. The control group showed no significant disorganization of the tumor tissues and no morphological changes in tumor cells. Conversely, the tumor tissues treated with Flat and MLA showed distinct cellular death in the irradiated regions as a result of photothermal effects (top row). According to TUNEL staining (bottom row), the Flat and MLA groups demonstrated apoptotic cell death in the treated lesions. However, it was noted that the Flat group was associated with smaller areas of irreversible thermal damage, leading to regeneration of the tumor tissue, even in the treated area. Consequently, the overgrowth of the tumor caused tumor hypoxia during the healing period (Figure 5a). Based on the pathologic scoring, the MLA group showed more significant histological responses (severe thermal injury and positive reaction of TUNEL staining) than the control (*p* < 0.005) and Flat groups (*p* < 0.05; Figure 5e).

## 4. Discussion

Ablative fractional laser (AFL) treatment has been widely studied for skin cancer therapies by removing the entire epidermis in the targeted area [24,25]. Several previous AFL studies reported the feasible inhibition of tumor growth at an early stage of cancer or dysplasia [26,27]. However, AFL treatment is often limited to the mere removal of the epidermis, which is unable to reach deeply or widely positioned tumor tissue [15]. Therefore, spatially enhanced distributions of optical energy during laser treatment are pivotal in attaining complete tumor inhibition and suppression of tumor recurrence. The current study verified the feasibility of MLA-assisted PTT with collective microthermal effects on tumors both by simulation and experiment, in comparison to Flat-based PTT. Given the same irradiation conditions, MLA produced a deeper and wider temperature development than Flat, indicating that it can develop high-irradiance micro-beams during irradiation [14]. In fact, the applied irradiance of each MLA-induced micro-beam was 72 mW/mm^2^ (72 mW/mm^2^ = 2 W/ (145 micro-beams and 96-μm^2^ micro-beam area)), whereas the irradiance of the Flat-induced macro-beam was 20 mW/mm^2^ (2 W/50.24 mm^2^ macro-beam area). Thus, by applying the collective thermal effects from a higher density of the micro-beams during the treatment (Figure 2 and Figure 3), the MLA-assisted irradiation widely generated irreversible thermal damage in the entire tumor tissue (Figure 5). In contrast, on account of a Gaussian-shaped beam profile, distributions of Flat-induced temperature became centric, which resulted in non-uniform and narrow treatment that is suitable for treating small and shallow tumors (Figure 2 and Figure 3; [28]). Therefore, the MLA-enhanced PTT exhibits therapeutic potential of achieving fractional treatment of wide and deep tumors by developing a more uniform and wider distribution of thermal energy after delivery of the micro-beams at high fluences for fractional cancer treatment.

The Flat group demonstrated that seven days after PTT, the tumor volume started to increase drastically, and at D 14, tumor necrosis occurred on the tumor surface (Flat) due to abnormal and uncontrollable regrowth (Figure 5c). The incomplete tumor treatment with non-uniform beam distributions from Flat could be responsible for stimulation of tumor growth and generation of the eventual necrosis [28]. Recently, a number of studies have reported rapid cancer cell proliferation and tumor growth as a result of laser stimulation [29,30]. Bamps et al. tested a 830 nm laser wavelength on head and neck squamous cell carcinoma and found cell proliferation with upregulation of phospho-protein kinase B (akt), phosphor-ERK, and Ki67 markers, evidencing the facilitated cancer aggressiveness [31]. Various studies also demonstrated that photobiomodulation with low laser light could proliferate cancer cells, including anaplastic thyroid cancer, oral carcinoma, and cervical cancer [32,33,34]. In addition, as the tumor grows excessively, tumor hypoxia can occur due to the lack of oxygen and insufficient blood supply [35]. The abnormal and rapid proliferation of the tumor can easily outgrow from marginal vasculature, decreasing the oxygen level to below 2 %. Consequently, the occurrence of tumor hypoxia leads to tumor necrosis [35,36]. Therefore, the marginal cancer cells after laser treatment can undergo photobiomodulative stimulation and eventually increase cell proliferation and cancer aggressiveness. Hence, to ensure treatment safety, adverse effects of PTT must be validated with a wide range of laser parameters (irradiance, irradiation time, and beam spot size) in in vivo cancer models in terms of cell proliferation, extent of complete tumor removal, and correlation with tumor regrowth.

Although MLA yielded more uniform and wider distributions of temperature on the tumor compared to Flat, the micro-beams emitted from MLA were still distributed in a Gaussian profile owing to their inherent optical nature (Figure 3). In turn, the uneven distribution of different micro-beam energy levels may lead to incomplete eradication of peripheral regions, particularly in large tumors. To achieve a more flat-top beam distribution consisting of comparable micro-beam energy on the tumor surface, diffractive optical elements (DOE) can be employed as an alternative to MLA for effective PTT. Unlike MLA with a Gaussian profile, DOE can yield flat-top distributions of the micro-beams by means of a homogenization process that overlaps the diffraction patterns [37]. Although energy loss (~20%) occurs during the homogenization process, DOE is hardly affected by fluctuations in the applied laser power and thus can deliver more uniformly stable micro-beams to the target than MLA. Therefore, further investigations will examine the feasibility of DOE-assisted PTT for effective cancer treatment in comparison to the current findings.

The current study used murine colon cancer cells (CT26) to perform preclinical validations of MLA-assisted PTT on tumors. The rapid growth and rich blood supply of CT26 cells in an in vivo xenograft model can emulate the pathological characteristics of human colon carcinoma [4,38]. Further studies will examine various cancer cells, such as breast cancer, liver cancer, and pancreatic cancer, to validate the therapeutic capacity of the MLA-assisted PTT in metastatic cancer and confirm acute and chronic responses of wound healing in the treated lesions. Furthermore, the current study selected a wavelength of 1064 nm and the IR1061 dye to facilitate photothermal effects during laser irradiation. For clinical translation, treatment conditions for MLA-assisted laser irradiation must be further optimized with various laser wavelengths (ultraviolet, visible, and infrared) and photothermal agents (IR 780 dye, carbon nanomaterial, and gold nanoparticle) to ensure the efficacy and safety of the MLA-assisted PTT on tumors [5]. Additionally, the present study merely used IR1061 dye as an absorbing agent for tissue phantom. Therefore, a tissue-mimicking phantom mixed with both absorbing and scattering agents should be tested to elucidate the distribution of optical fluence [39].

## 5. Conclusions

The current study demonstrates the feasibility of MLA-assisted PTT for the effective treatment of colon cancer on in vivo murine models. Collective photothermal effects from MLA-induced micro-beams account for the spatial enhancement of thermal damage in the irradiated tumor, as well as complete tumor treatment without recurrence. Future studies are expected to investigate the proposed therapeutic capacity with various wavelengths and photothermal agents to further warrant efficacy and safety of MLA-assisted PTT on cancer for clinical translation.

## Figures and Tables

**Table 1:** Summary of thermo-physical properties used for numerical simulation [18,19,20].

Parameters	Value
Absorption coefficient (μ_a_, mm^-1^)	1
Density (ρ, kg·mm^-1^)	1060
Thermal conductivity (k, W/m·K)	0.303
Specific heat (C_p_, J/kg·K)	3600
Beam radius (r_0_, mm)	8
Convective heat coefficient (h, W/m^2^·K)	10
Air temperature (T_air_, K)	293.15
Laser power (P, W)	1

## Supplementary Materials

The following are available online at https://www.mdpi.com/2072-6694/13/5/1146/s1, Figure S1: Comparison of temperature rises measured from PBS and IR 1061 aqueous solution with various concentrations (0 for PBS, 100, 200, and 300 µg/mL) after laser irradiation at three irradiances (1, 2, and 3 W/cm2) for 180 s, Figure S2: Infrared thermographic compilations of laser irradiation on IR 1061 solution with Flat and MLA for 180 s (scale bar = 3 mm), Video S1: Temperature developments of tumor during laser irradiations with Flat and MLA (2 W/cm2).
