# Supplementary material for: Multi-Lens Arrays (MLA)-Assisted Photothermal Effects for Enhanced Fractional Cancer Treatment: Computational and Experimental Validations
Source: Cancers (Basel). 2021 Mar 8;13(5):1146. doi: 10.3390/cancers13051146 (PMC7962441; doi:10.3390/cancers13051146)
Supplement: Supplementary file 1 [file cancers-13-01146-s001.zip › cancers-1115498supp-conversion/cancers-1115498 -Supplementary template.docx]

Supplementary Materials: Multi-lens Arrays (MLA)-assisted Photothermal Effects for Enhanced Fractional Cancer Treatment: Computational and Experimental Validations

Hyejin Kim , Hanjae Pyo , Hyeonsoo Kim and Hyun Wook Kang


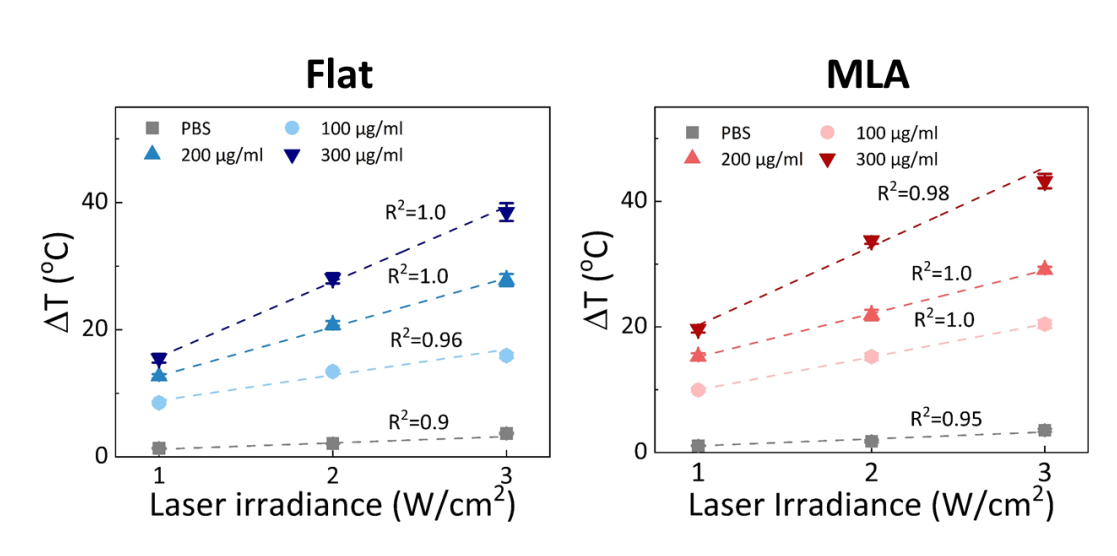


**Figure S1.** Comparison of temperature rises measured from PBS and IR 1061 aqueous solution with various concentrations (0 for PBS, 100, 200, and 300 µg/mL) after laser irradiation at three irradiances (1, 2, and 3 W/cm^2^) for 180 s.


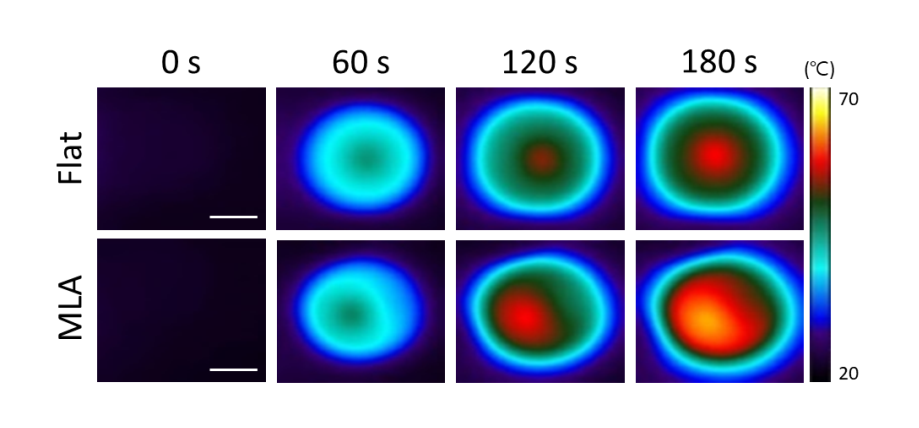


**Figure S2.** Infrared thermographic compilations of laser irradiation on IR 1061 solution with Flat and MLA for 180 s (scale bar = 3 mm).
